# Comparison Stratagems of Post-Screening Management of Anti-HCV-Positive Community Residents: Simple Notification, Active Referral, or Accessible Medical Care

**DOI:** 10.1371/journal.pone.0126031

**Published:** 2015-05-13

**Authors:** Yuan-Hung Kuo, Pao-Fei Chen, Jing-Houng Wang, Kuo-Chin Chang, Kwong-Ming Kee, Ming-Chao Tsai, Chun-Yin Lin, Sheng-Che Lin, Lin-San Tsai, Shu-Chuan Chen, Sheng-Nan Lu

**Affiliations:** 1 Division of Hepato-Gastroenterology, Department of Internal Medicine, Kaohsiung Chang Gung Memorial Hospital and Chang Gung University College of Medicine, Kaohsiung, Taiwan; 2 Health Center of Yanpu Township, Pingtung, Taiwan; 3 Health Center of Yujing district, Tainan, Taiwan; 4 Department of Health, Tainan City Government, Tainan, Taiwan; National Taiwan University Hospital, TAIWAN

## Abstract

To elucidate the results of post-screening care stratagems for anti-hepatitis C virus (HCV)-positive subjects in the community. Part I methods: The intervention program: A total of 151,790 subjects underwent a large-scale healthcare screening. Subjects aged less than 65 years, with anti-HCV-positive and alanine aminotransferase (ALT) level more than 80 IU/L were followed-up to answer a structured questionnaire. Those responders who met the reimbursement criteria of Taiwan’s National Health Insurance for anti-HCV treatment were referred for treatment. Part II: The accessible medical care program: In Yujing township, 271 HCV residents who have been screened before were invited to a bi-weekly hepatitis clinic in Yujing health center. Part-I results: A total of 907 anti-HCV-positive subjects responded and 197(21.7%) were advised the treatment, but only 83(9.2%) did. Finally, 47 patients achieved a sustained virological response (SVR). After this intervention program, 96(10.6%) additional patients were encouraged to be referred, 33(3.6%) received treatment and 20 obtained a SVR. Part II: A total of 140(51.7%) subjects responded and 112 were anti-HCV-positive including 31(27.7%) HCV RNA-negative, 49(43.8%) HCV RNA-positive plus ALT less than 40 IU/L and 32(28.5%) HCV RNA-positive plus ALT more than 40 IU/L. During the follow-up, 14 of 49 patients had ALT more than 40 IU/L. Among 46 eligible HCV patients, 15(32.6%) received treatment and 10 achieved a SVR. Simple notification only made 9.2% of the screened HCV patients treat. Active referral could encourage additional 3.6% to be treated. Additionally, accessible medical care program could result in treatment of 32.6% elderly eligible patients.

## Introduction

Approximately 170 million patients are chronically infected with hepatitis C virus (HCV) worldwide [1]. Chronic hepatitis C (CHC) is one of the leading causes of development of liver cirrhosis and hepatocellular carcinoma (HCC) [2]. Deaths related to the complications of liver cirrhosis occur at an incidence of approximately 4% per year, whereas HCC occurs at an estimated incidence of 1–5% per year in this population [3]. Successful eradication of HCV with antiviral treatment, such as intramuscular peg-interferon plus oral ribavirin regimen or all-oral direct-acting antiviral agents (DAA) could reduce the risk of developing liver cirrhosis or HCC and improve the long term survival in CHC patients [4–9]. Hence, early detection of the HCV patients suitable for antiviral treatment is an appropriate preventive strategy to reduce mobility and mortality.

In Taiwan, the HCV prevalence is approximately 4.4% and nearly 1 million people are infected [10]. Several county- or city- Public Health Bureaus conduct annual community health screenings in Taiwan, including HCV screening. Although many anti-HCV positive subjects were detected in these hepatitis screenings, post-screening care to treat these patients was not very efficient; most authorities only informing the screened patients of the test results. Some patients were educated for hepatitis management and advised further clinic visits; however, most screened CHC patients did not get an active referral for antiviral treatment. Consequently, there was still a gap between the screened CHC patients in the community and the treated patients in the medical institutes; even though current standard of care (SOC) including peg-interferon plus ribavirin regimen has been reimbursed by the National Health Insurance (NHI) in Taiwan since 2003 [11]. The HCV patients meeting the treatment criteria such as positive anti-HCV, two values (three months apart) of abnormal alanine aminotransferase (ALT) levels higher than two times of upper limit of normal (ULN) and liver biopsy proved hepatic fibrosis could be covered by the reimbursement policy. In view of the high eradication rate of treatment in Asian HCV patients, reimbursement criteria were loosened in 2009 to meet the current international guidelines [12]. The revised criteria included positive anti-HCV, once abnormal ALT level higher than ULN and HCV RNA-positive, which shortened the waiting period for treatment and avoided the possible complications of liver biopsy.

To elucidate the post-screening medical care condition of the CHC patients, this study was conducted with two post-screening interventions to show the treatment rates among the different strategies, including simple notification, active referral and establishment of an accessible medical care.

## Methods

### Ethics statement

This study was approved by the Institutional Review Board (IRB) of Kaohsiung Chang Gung Memorial hospital and a written informed consent was obtained from each participant. This study was conducted according to the principles expressed in the Declaration of Helsinki and the consent procedure in the study was also proved by the IRB of Kaohsiung Chang Gung Memorial Hospital.

### Study background

Tainan County is located in southern Taiwan with 533 villages, spread in 31 townships. The population of Tainan County is approximately 1.1 million, and 42% of the population is older than or equal to 40 years of age. Since 2004, an annual county-wide comprehensive community health examination is being conducted by the Public Health Bureau of Tainan County for residents older than 40 years of age [13–17]. Residents aged 40 years and above could participate in this series of screening once in every three years, whereas those above 65 years of age could participate every year.

### Part I: The experimental intervention program

#### Study area and patients

A total of 151,790 residents, including 55,571(36.6%) males and 96,219 (63.4%) females, aged 40 years and above, with a mean age of 59.9±12.7 years, were involved in the county-wide community screening from 2004 to 2007 in Tainan. Blood samples were obtained for examining hepatitis B surface antigen (HBsAg) and anti-HCV, biochemical tests, complete blood count and alpha-fetoprotein. All participants received a documented report of test results. In 2007, the subjects, aged less than 65 years, with anti-HCV-positive and ALT level more than two times of ULN were followed-up to answer a structured questionnaire, which inquired their medical status after the community hepatitis screening and notified them to return to local health centers for re-checking their ALT levels.

#### Referral policy and NHI reimbursement criteria for anti-HCV treatment

In 2007, the reimbursement criteria of anti-HCV treatment of NHI included positive anti-HCV, two values (three months apart) of abnormal alanine aminotransferase (ALT) levels higher than two times of upper limit of normal (ULN) and liver biopsy proved hepatic fibrosis (Table 1) [11]. The patients with two abnormal ALT levels were actively referred to the nearby gastroenterologists of local or regional hospitals for the assessment of antiviral treatment including liver biopsy. The suitable cases could then receive SOC with intramuscular peg-interferon plus oral ribavirin regime according to the treatment guidelines [18,19].

**Table 1:** The promulgation years of reimbursement criteria for treatment of HCV of the National Health Insurance in Taiwan and our intervention studies.

	Reimbursement criteria of HCV treatment	Our intervention studies
Nov, 2003	Anti-HCV(+); twice ALT≧80 IU/L in three	
	months apart within half a year; hepatic	
	fibrosis grade≧ F1 level*	
Nov, 2007		Experimental intervention
		program
Nov, 2009	Anti-HCV(+); once ALT≧40 IU/L; hepatic	
	fibrosis grade≧F1 level or HCV RNA(+)	
Mar, 2011		Accessible medical care
		program

*The fibrosis grade was according to the Metavir scoring system: F0: no scaring; F1: minimal scarring; F2: scarring has occurred and extends outside the areas in the liver that contains blood vessels; F3: bridging fibrosis is spreading and connecting to other areas that contain fibrosis; F4: cirrhosis or advanced scarring of the liver.

Abbreviation: HCV: hepatitis C virus; ALT: Alanine Aminotransferase.

### Part II: The accessible medical care program

#### Study area and patients

Yujing is one of the mountain townships in Tainan with a population of approximately 16,000 and an estimated HCV prevalence of 8% [13]. From 2004 to 2010, in the county-wide health screenings, a total of 271 residents were diagnosed with positive anti-HCV. In March 2011, these residents were invited to this accessible medical care program by mail or telephone. All participants underwent anthropometric measurements, blood examination including biochemistry and virological tests, ultrasound and Fibroscan examinations. Those with negative anti-HCV status were excluded from this program. The participating patients were managed in a special hepatitis clinic in Yujing health center, conducted by two hepatologists bi-weekly. The interval of follow-up was three months for cirrhotic patients and six months for the others.

#### Revised NHI reimbursement criteria of anti-HCV treatment

The reimbursement criteria of anti-HCV treatment of Taiwan NHI was revised in 2009,^12^ (Table 1) which was simplified to positive anti-HCV, once abnormal ALT level higher than ULN and HCV RNA-positive. Positive anti-HCV subjects who met the reimbursement criteria could receive SOC regimens in Yujing hepatitis clinic according to the treatment guidelines [20,21].

### Laboratory Examinations

Serum ALT level was measured by a Hitachi 747 auto-analyzer (Hitachi Ltd.,Tokyo, Japan). In 2004, anti-HCV test was assessed by ELISA utilizing a SP-NANBASE C-96 3.0 plate (General Biological Corp.,Hsinchu, Taiwan). From 2005 to 2011, the anti-HCV test was assessed utilizing Abbott AxSYM HCV 3.0, and the Micro-Enzyme-Immunoassay Analysis (MEIA) was conducted (Abbott, IL, USA). Levels of HCV RNA were detected using a Cobas AmpliPrep/Cobas TaqMan HCV kit (Amplicor, Roche Diagnostics, Branchburg, NJ), with a lower limit of detection of 15 IU/ml.

### Statistical Analysis

Logistic regression analysis was used to explore the predictors associated with active referral and treatment in the experimental intervention study. A probability lower than 0.05 level was defined as statistically significant. Statistical analysis was performed using SPSS 15.

## Results

### Part I: The experimental intervention program

#### Baseline characteristics of examined subjects

The baseline characteristics of 151,790 subjects participating in this comprehensive community screening are shown in Table 2. Among these participants of community screening, 971 eligible subjects with positive anti-HCV, ALT more than two times of the ULN and age less than 65 years were followed-up. Out of these 971 subjects, a total of 907 (93.4%), including 374 males and 533 females with a mean age of 56±6.3 years responded. Among them, 100 (11%) subjects were both HBsAg and anti-HCV positive.

**Table 2:** Basic information of the subjects in community-based comprehensive health screening in Tainan County from 2004 to 2007.

	Total subjects n = 151790	Eligible CHC subjects* n = 907
Age (years, mean±SD)	59.9±12.7	56.4± 6.3
Sex, n (%)		
Male	55571 (36.6)	374(41.2)
Female	96219 (63.4)	533(58.8)
Hepatitis etiology, n (%)		
HBsAg positive	16776 (11.1)	0
Anti-HCV positive	13880 (9.1)	807(89)
HBsAg and anti-HCV positive	1601 (1.1)	100(11)
HBsAg and anti-HCV negative	119533 (78.7)	0
ALT level (IU/L)		
≦40	131270 (86.5)	0
40–80	15476 (10.2)	0
80–200	4174 (2.7)	814(89.8)
>200	870 (0.6)	93(10.2)

*Eligible CHC subjects: the subjects with Age<65 years, anti-HCV positive, and ALT≧80 IU / L.

Abbreviation: CHC: chronic hepatitis C; HCV: hepatitis C virus; HBsAg: hepatitis B virus surface antigen; ALT: Alanine Aminotransferase; SD: standard deviation

#### Medical status after the screening

**Fig 1:**
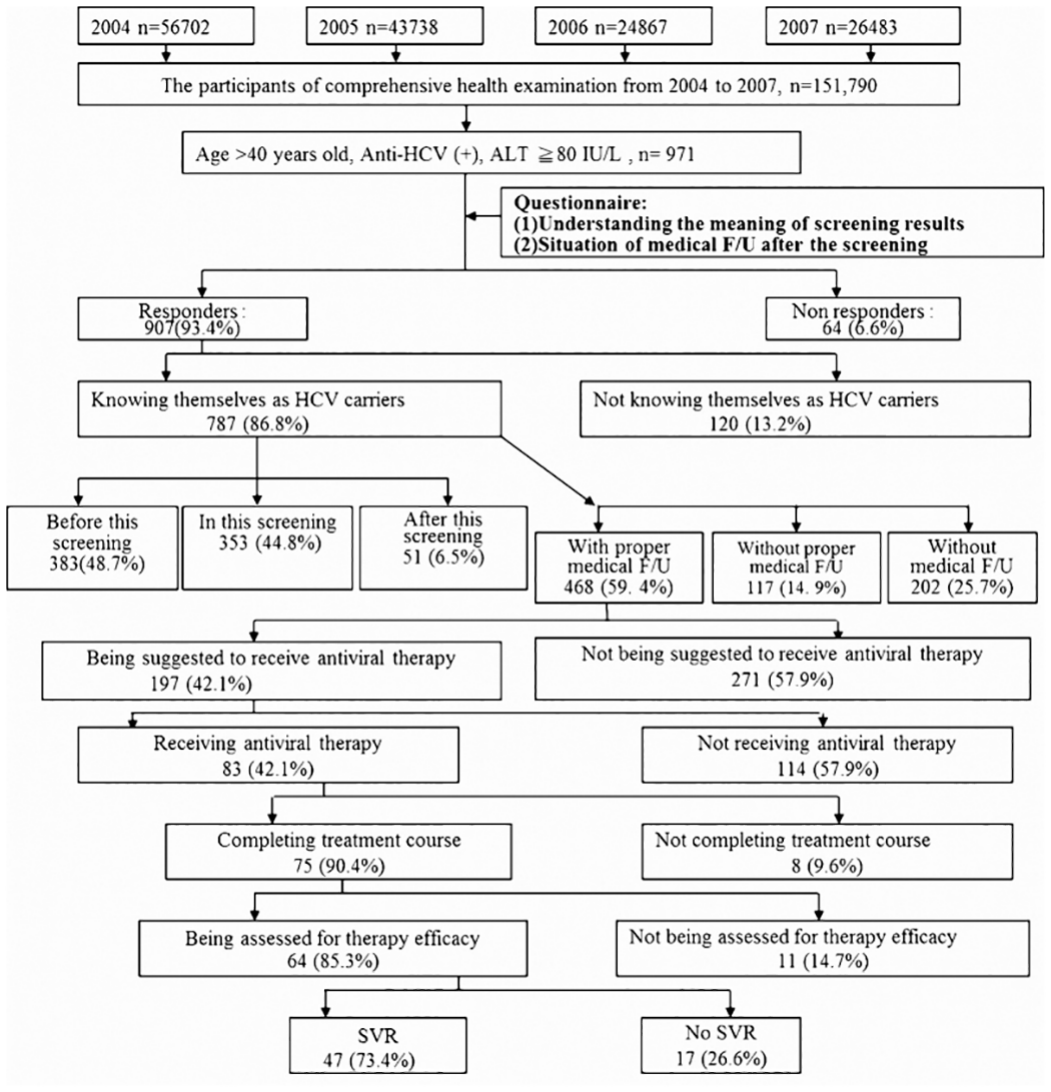
The situation of medical follow-up of anti-HCV(+) patients after hepatitis screening.

As per the questionnaires’ analysis, out of 907 eligible anti-HCV positive patients, only 787 (86.8%) were aware that they were infected (Fig 1). Among these patients, 353 (44.8%) received this information from this screening and 468 (59.4%) received the information on visiting a hepatitis clinic with regular follow-up after the screening.

Among those 468 patients with proper medical care, 197 (42.1%) were advised to receiv anti-HCV treatment with peg-interferon plus ribavirin, but only 83 (42.1%) received the treatment. As per the treatment results, among the 64 patients who completed the treatment and were well assessed, 47 (73.4%) patients finally obtained a sustained virological response (SVR).

#### The implementation of the experimental intervention program

**Fig 2:**
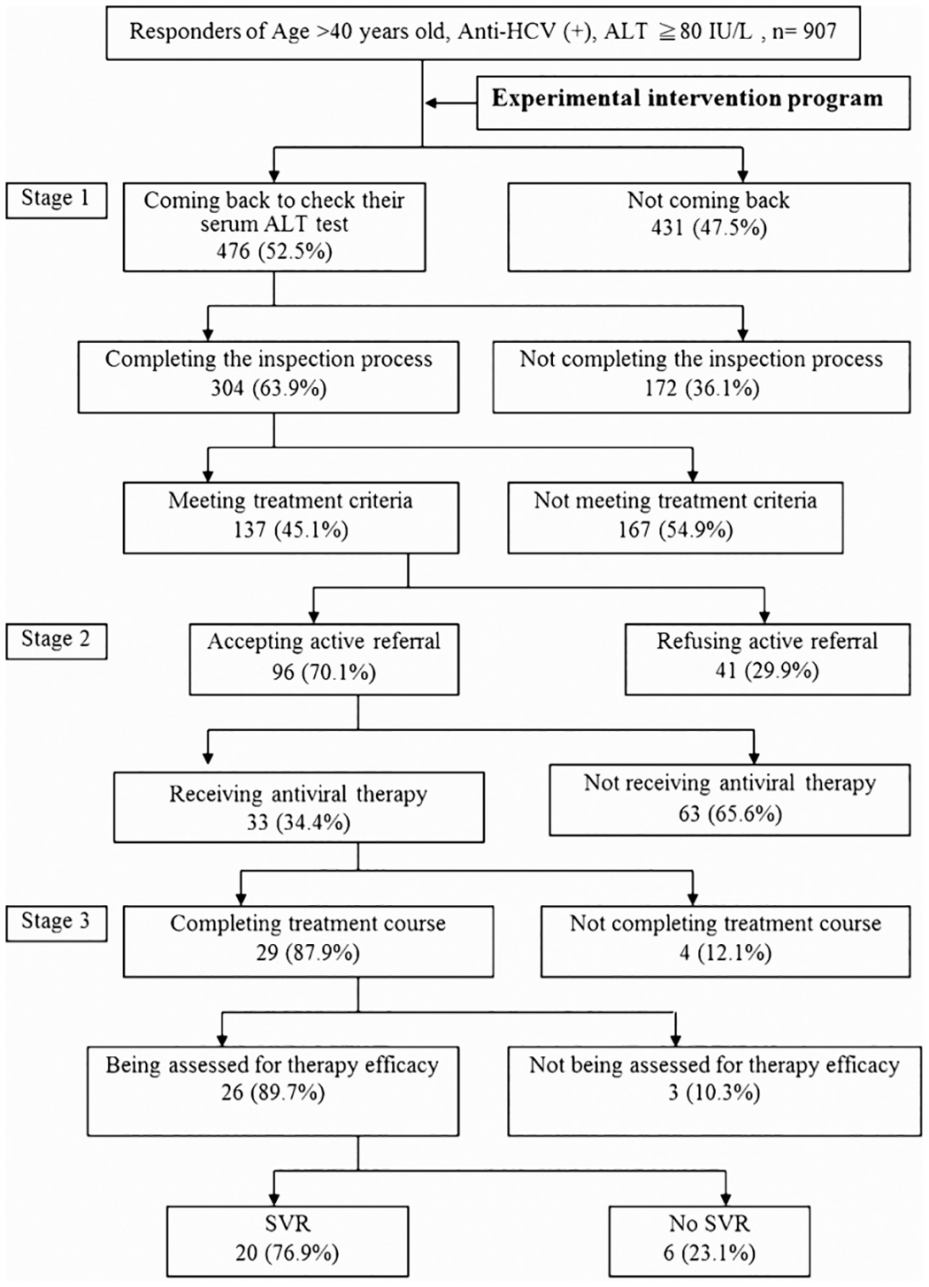
The results of the experimental intervention program. Stage1: The evaluation of the criteria of anti-HCV treatment; Stage 2: Active referral of eligible anti-HCV(+) patients for treatment; Stage 3: The outcome of anti-HCV treatment.

A total of 907 HCV patients were notified to check the serum ALT levels at local health centers; 476 (52.5%) returned to take the first ALT test (Fig 2). Among these 476 patients, 304 (63.9%) completed the process of blood test held twice, three months apart. 137 (45.1%) patients had two ALT levels more than 80 IU/L, which met the reimbursement criteria of NHI for anti-HCV treatment in 2007.

Among the 137 eligible HCV patients, 96 (70.1%) accepted the “Active Referral Program” to the nearby medical institutes for the evaluation of anti-HCV treatment. Additional 33(34.4%) patients agreed to receive anti-HCV treatment with peginterferon plus ribavirin and 29 completed the treatment. Among 26 patients with complete assessment, 20 (76.9%) finally obtained SVR.

#### Associated factors with the implementation of the experimental intervention program

Multivariate analysis indicated that factors, such as male gender (p = 0.01), patients who did not have clinical visit after the screening (p<0.001) and those who did not receive proper medical care after the screening (p<0.001) were associated with the execution of the caring activities (Table 3).

**Table 3:** Impact of experimental intervention program of association factors analysis.

Subgroups	Associated factors	No	Uni-variate OR(95%CI)	p-value	Multi-variate OR(95%CI)	p-value
Receiving ALT test (n = 907)	Age (years old)					
	≦55years	383	1	0.177		
	>55years	524	0.82 (0.63–1.07)			
	Sex					
	Female	533	1	0.001	1	0.01
	Male	374	1.55 (1.18–2.02)		1.43 (1.08–1.91)	
	Knowing being infected with HCV					
	Yes	787	1	0.002		
	No	120	1.89 (1.27–2.83)			
	Having hepatitis clinic visit					
	Ever	645	1	<0.001	1	<0.001
	Never	262	3.15 (2.30–4.29)		1.59 (1.12–2.28)	
	Under proper medical care*					
	Yes	350	1		1	<0.001
	No	557	4.36 (3.28–5.81)	<0.001	3.49(2.52–4.82)	
Receiving repeat ALT test (n = 476)	Age (years old)					
	≦55years	212	1	0.221		
	>55years	264	0.75 (0.52–1.10)			
	Sex					
	Female	256	1			
	Male	220	1.06 (0.73–1.54)	0.775		
	Knowing being infected with HCV					
	Yes	397	1	0.345		
	No	79	1.89 (0.55–1.49)			
	Having hepatitis clinic visit					
	Ever	288	1	0.425		
	Never	188	1.04 (0.71–1.52)			
	Under proper medical care*					
	Yes	108	1	0.021	1	0.035
	No	368	1.57 (1.02–2.44)		1.57 (1.02–2.44)	
Receiving active referral for medical treatment (n = 137)	Age (years old)					
	≦55years	61	1	0.596		
	>55years	76	1.98 (0.47–2.04)			
	Sex					
	Female	74	1	0.596		
	Male	63	1.98 (0.47–2.04)			
	Knowing being infected with HCV					
	Yes	120	1	0.256		
	No	17	2.16 (0.59–7.97)			
	Having hepatitis clinic visit					
	Ever	87	1	<0.001	1	<0.001
	Never	50	0.36 (0.17–0.76)		0.36 (0.17–0.76)	
	Under proper medical care*					
	Yes	42	1	0.025		
	No	95	0.44 (1.02–2.44)			
Receiving antiviral therapy (n = 96)	Age (years old)					
	≦55years	41	1	0.68		
	>55years	55	0.23 (0.52–2.90)			
	Sex					
	Female	52	1	0.575		
	Male	44	0.98 (0.42–2.28)			
	Knowing being infected with HCV					
	Yes	82	1	0.022		
	No	14	0.12 (0.15–0.96)			
	Having hepatitis clinic visit					
	Ever	68	1	<0.001	1	0.026
	Never	28	0.042(0.05–0.34)		0.09(0.01–0.75)	
	Under proper medical care*					
	Yes	34	1	0.035	1	0.004
	No	62	0.12 (0.02–0.44)		0.38(0.22–0.76)	

*Under proper medical care: Receiving regular follow-up by the hepatologists.

Abbreviation: No: number; OR: odds ration; CI: confidence interval; ALT: Alanine Aminotransferase; HCV: hepatitis C virus.

Regarding the acceptance of the active referral, multivariate analysis indicated that those who did not have clinical visit after the screening had a lower acceptance rate of active referral than those who did the clinical visit [odds ratio (OR) = 0.36, p<0.001].

With respect to the outcome of active referral, multivariate analysis showed that clinical visits after screening (*P* = 0.026) and proper medical care after screening (*P* = 0.004) are the independent factors associated with acceptance of antiviral treatment. It seemed that those patients who lacked regular medical follow-up after the screening inclined to receive the blood tests in local health centers but refused the active referral or antiviral treatment in the medical institutes.

### Part II: the accessible medical care program

**Fig 3:**
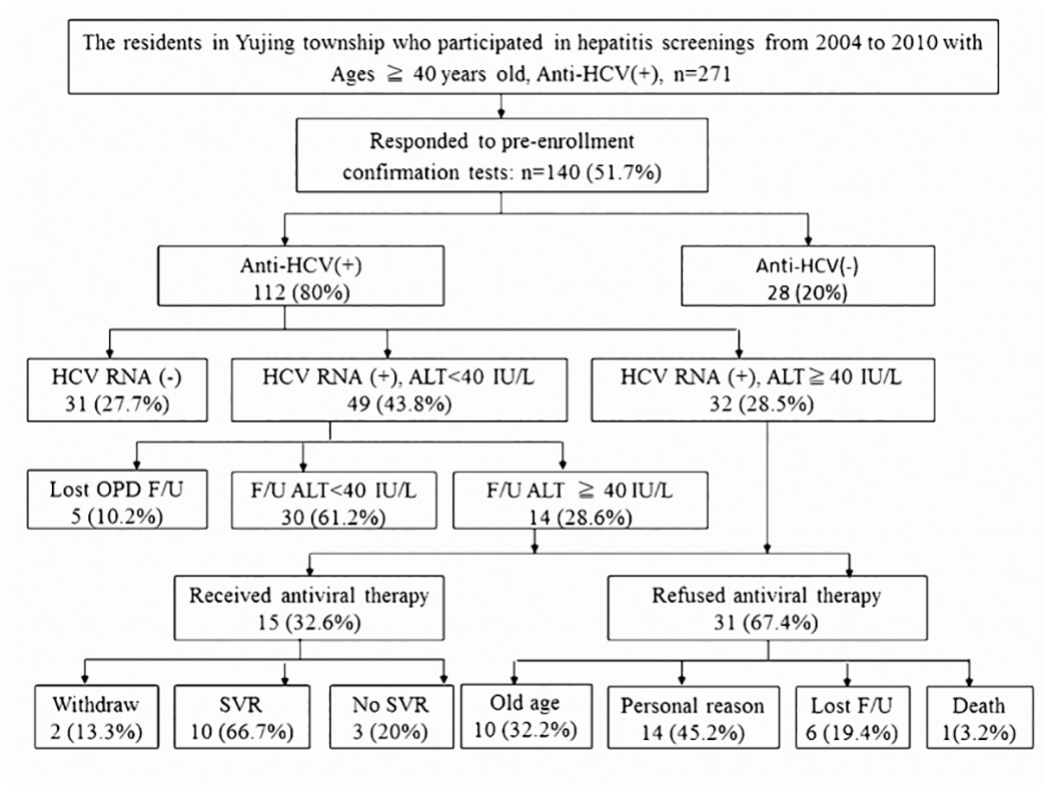
The results of the anti-HCV(+) patients after hepatitis screening in the accessible medical care program.

A total of 140 (51.7%) residents responding to this accessible medical care program in 2011, included 112 anti-HCV positive, and 28 anti-HCV negative subjects (Fig 3) Among 112 anti-HCV positive subjects, 31 (27.7%) were HCV RNA-negative, 49 (43.8%) were HCV RNA-positive plus ALT level less than 40 IU/L and 32 (28.5%) were HCV RNA-positive plus ALT level more than 40 IU/L; among which the last group met the revised reimbursement criteria of NHI. During the bi-weekly follow-up at Yujing health center, 14 of the 49 HCV-RNA positive patients had an elevated ALT of more than 40 IU/L. Among those 46 CHC patients who met the reimbursement criterial of NHI, 15 (32.6%) underwent SOC with peginterferon plus ribavirin at the clinic. 2 patients withdrew the treatment, whereas 13 patients completed the treatment; out of which 10 finally achieved the SVR. In the other 31 patients for treatment, 1 patient died, 6 lost on follow-up, 14 refused treatment due to personal reasons such as living alone or fear of treatment related side effects, and 10 were contraindicated for treatment due to old age (mean age: 76.1± 6.3 years, range:72.2–81.4 years).

### Medical status of the same HCV subjects in Part I and Part II intervensions

There were 18 post-screened HCV eligible subjects in Yujing township responded the questionnaire in 2007 (Table 4). Among them, 4 (22.2%) subjects have been treated after the screening (2 had SVR), and 7 (38.9%) came back for the intervention study. Among 7 subjects participated into Part I intervention, 5 met the past Taiwan National Health Insurance (NHI) treatment criteria; 4 received the active referral to the neighboring medical institutes but none of them received the treatment finally. In 2011, 8 (44.4%) of these 18 eligible cases participated into the Part II accessible medical care program. Among them, one has been treated before 2007 and obtained SVR; 5 of the other seven cases met the new NHI criteria and 3 received anti-HCV treatment during the special hepatitis clinic in Yujing and all obtained SVR. In particular, all 5 cases met the past NHI criteria in 2007 participated into the part II study in 2011 and 2 received anti-HCV treatment finally.

**Table 4:** Medical status of the 18 HCV eligible cases in Yujing township.

No	Sex	Age in 2007	Treated experienced before 2007	Met NHI guideline in 2007	Active referral in 2007	Treated in 2007	Accessible care in 2011	Met new NHI guideline in 2011	Treated in 2011
1	M	56	(-)	(+)	(+)	(-)	(+)	(+)	(-)
2	M	54	(-)	(+)	(+)	(-)	(+)	(+)	(-)
3	M	63	(-)	(-)	/	/	(+)	(+)	(-)
4	F	60	(-)	(+)	(+)	(-)	(+)	(-)	/
5	F	44	(-)	(+)	(-)	/	(+)	(+)	(+)*
6	F	59	(+)*	/	/	/	(+)	(-)	/
7	F	60	(-)	(+)	(+)	(-)	(+)	(+)	(+)*
8	F	57	(-)	(-)	/	/	(+)	(+)	(+)*
9	M	57	(+)	/	/	/	/	/	/
10	M	43	(-)	/	/	/	/	/	/
11	M	62	(-)	/	/	/	/	/	/
12	F	55	(-)	(-)	/	/	/	/	/
13	F	57	(+)*	/	/	/	/	/	/
14	F	63	(-)	/	/	/	/	/	/
15	F	47	(-)	/	/	/	/	/	/
16	F	62	(-)	/	/	/	/	/	/
17	F	59	(+)	/	/	/	/	/	/
18	F	62	(-)	(-)	/	/	/	/	/

(-) represents negative result; (+) represents positive result; /:represents patient didn`t do the exam or receive the treatment.

*Patients obtained SVR after antiviral treatment.

Abbreviation: HCV: hepatitis C virus; NHI: National Health Insurance; M: male; F: female.

## Discussion

The aim of part I of this current study was to elucidate the results of different post-screening care models in screened HCV patients, including simple notification of the screening results and active referral. The current study indicated that despite dispatching the screening report to the screened HCV patients, 120 (13.2%) of them still did not know that they have been infected with HCV. This was similar to the result of another large-scale community screening in Taiwan [22], which showed that many subjects participated in the hepatitis screening but did not confirm whether they were HBV or HCV carriers after the screening. In addition, the current study also found that among those 787 (86.8%) self-recognized HCV patients, 48.7% already identified that they were infected before participating in this hepatitis screening. Therefore, to avoid the repeated efforts and resources on repeat screening, HBV or HCV carriers should be taken into consideration by the government in making the future screening policy.

It was also found that 197(21.7%) of all eligible CHC patients have been advised to receive anti-HCV treatment, but only 83 (9.2%) patients received the treatment. In the current study, these screened HCV patients were called back for follow-up in local health centers and tried to find more suitable cases for anti-HCV treatment. For the patients who met the criteria of treatment, 96 (70.1%) patients accepted the active referral to the neighboring medical institutes for treatment. Finally, additional 33 (3.2%) of 907 eligible HCV patients received treatment and after post-treatment completion assessment, 20 (76.9%) obtained a SVR. To those screened HCV patients who were unaware of being infected or inactive to their infection, active referral could educate them about the illness and encourage them to receive active treatment.

After the multivariate analysis, we found those HCV patients without clinical visit or proper medical care intended back to receive complete ALT tests in local health centers. However, the same group was unwilling to receive active referral or anti-HCV treatment in the medical institutes. It reflected that most of the untreated HCV patients might agree to receive the local examinations but refuse the referral treatment. In addition, the review article about best strategies for global HCV eradication has also indicated that effective linkage of screening and treatment was important to increase HCV eradication rate [23]. Hence, we conducted the part II study to elucidate the role of accessible medical care model post-hepatitis screening in the rural area. In the initial examination, we found that 27.7% of elderly anti-HCV positive patients were HCV-RNA negative and did not require further treatment. Indeed, except those who had liver cirrhosis, this stable group might not need a long time regular follow-up. Otherwise, a regular monitor of the hepatitis status was still necessary for the other 72.3% of elderly CHC patients. Among HCV patients suitable for treatment, 15(32.6%) received anti-HCV treatment with SOC regimes in the local health center. Among other 31 candidates for treatment, 10 were contraindicated for current peg-interferon based treatment due to old age (mean age: 76.1± 6.3 years, range: 72.2–81.4 years). Oral DAA might be beneficial to these elderly patients. Regarding the treatment effect, 10 (66.7%) patients achieved a SVR, which was comparable with the previous reports in Taiwan [24–26].

Although the Taiwan NHI treatment criteria was loosened since 2009 to make suitable cases easier to treat, some elder patients still disfavored inaccessible treatment, especially in the rural area. For example, in Yujing township, the nearest hospital for antiviral treatment is at least 30 kilometers far away. In the part I study, 5 eligible cases in Yujing township were suited for referral, 4 cases received active referral but none of them received treatment. All these 5 cases participated into our part II study, 4 cases met the revised NHI treatment criteria and 2 received anti-HCV treatment and obtained SVR finally. Hence, in a rural area with higher HCV prevalence, to provide an accessible medical care could treat more eligible HCV patients. The implementation of this accessible model is feasible and will require the collaboration between government agencies, health care providers, and community organizations to make policy changes.

There were still some limitations. First, the factors associated with successful HCV eradication were complicated and might include host factor, virus condition, regimen effect, environment condition and care quality [27]. The current study tried to elucidate the status of screened HCV patients post-hepatitis screening but lacked the detailed information about the individual treatment. In addition, although we found post-screened HCV patients in the rural area could obtain a better care in this accessible medical care model and increase the acceptance rate for anti-HCV treatment; the cost effectiveness of this accessible medical care program is undetermined. Finally, this manuscript reported two real world interventions for post-screening HCV patients, but not a two comparison study.

In Taiwan, under the old anti-HCV treatment criteria of NHI, simple notification of the screening report after the screening only made 9.2% of screened CHC patients receive the treatment. Active referral could encourage additional 3.6% of patients to be treated. The currently revised treatment criteria of NHI make the treatment easier to receive. Among those screened elderly candidates suitable for treatment in the rural area, accessible medical care program could result in treating at least 32.6% of the patients.
